# Supplementary material for: A synthetic cell permeable antioxidant protects neurons against acute oxidative stress
Source: Sci Rep. 2017 Sep 19;7:11857. doi: 10.1038/s41598-017-12072-5 (PMC5605738; doi:10.1038/s41598-017-12072-5)
Supplement: Supplementary file 1 — Supplementary Information [file 41598_2017_12072_MOESM1_ESM.pdf]

Supplementary information for:

A synthetic cell permeable antioxidant protects neurons against acute oxidative stress

Nicola J. Drummond, Nick O. Davies, Janet E. Lovett, Mark R. Miller, Graeme Cook, Thomas Becker, Catherina G. Becker, Donald B. McPhail, Tilo Kunath

Supplementary Figure 1

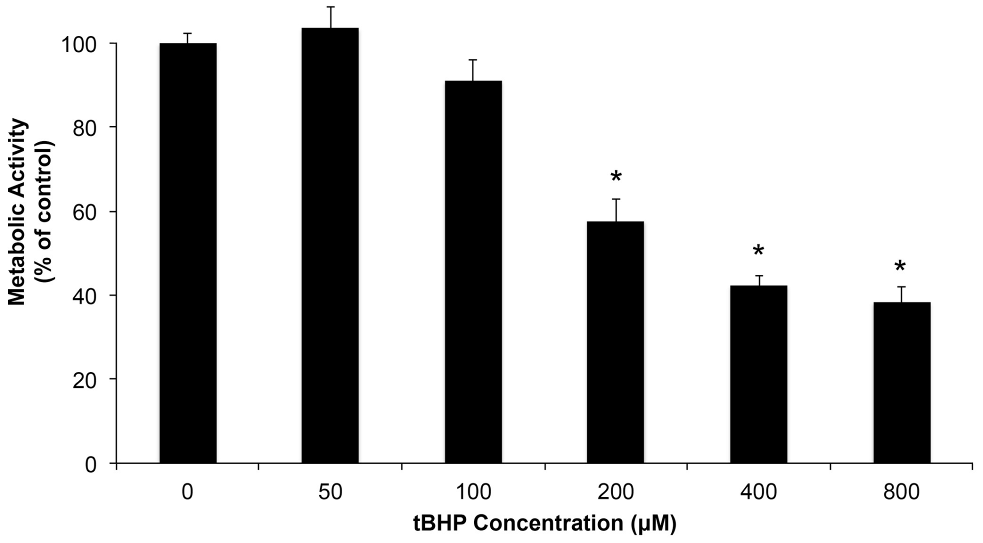

Supplementary Figure 1. *t*BHP-induced cellular toxicity. SH-SY5Y cells were incubated with various concentrations of *t*BHP for 5 hours with the MTS assay being performed in the last hour to assess cell viability. Bars represent the mean from 3 independent experiments (n=3), each with triplicate wells, error bars represent the SEM. ANOVA with a Tukey’s post-hoc test was performed to assess the significant difference between *t*BHP-treated cells and vehicle-treated cells, * $p<0.05$.

Supplementary Movie 1
Videomicroscopy of SH-SY5Y cells treated with 400 μM tBHP for 5h. Live cell imaging obtained with the Cell-IQ system. Cells exhibit morphological signs of lipid peroxidation and death.

Supplementary Movie 2
Videomicroscopy of SH-SY5Y cells treated with 400 μM tBHP in the presence of 1 μM Proxison for 5h. Live cell imaging obtained with the Cell-IQ system. Cells exhibit small amounts of membrane blebbing, but otherwise remain healthy and alive after 5h.

Supplementary Figure 2

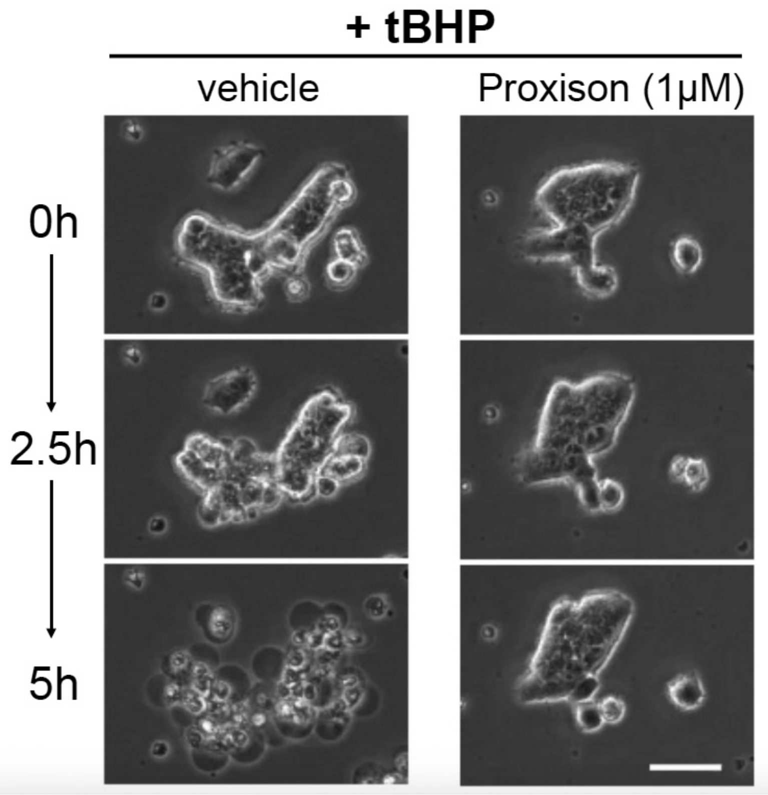

Supplementary Figure 2. *t*BHP treated cells in the absence or presence of Proxison. Images from Cell-IQ videomicroscopy of SH-SY5Y cells at 0h, 2.5h, and 5h. Proxison (1 μM) fully protects cells against a high-dose of *t*BHP (400 μM). Scale bar, 75 μm.

Supplementary Figure 3

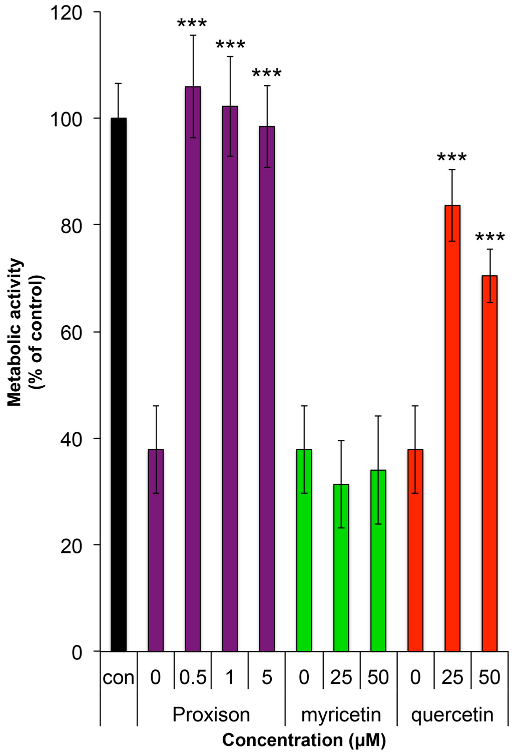

Supplementary Figure 3. *t*BHP-induced cellular toxicity is rescued by Proxison and quercetin in Neuroscreen-1™ rat cells. Neuroscreen-1 cells were incubated with 400 uM of *t*BHP for 5 hours in the absence or presence of varying concentrations of Proxison, myricetin, or quercetin. The MTS assay was performed in the last hour to assess cell viability. Bars represent the mean from 3 independent experiments. ANOVA with a Tukey's post-hoc test was performed to assess the significant difference between *t*BHP-treated cells and antioxidant-treated cells, *** $p<0.001$.

Supplementary Figure 4

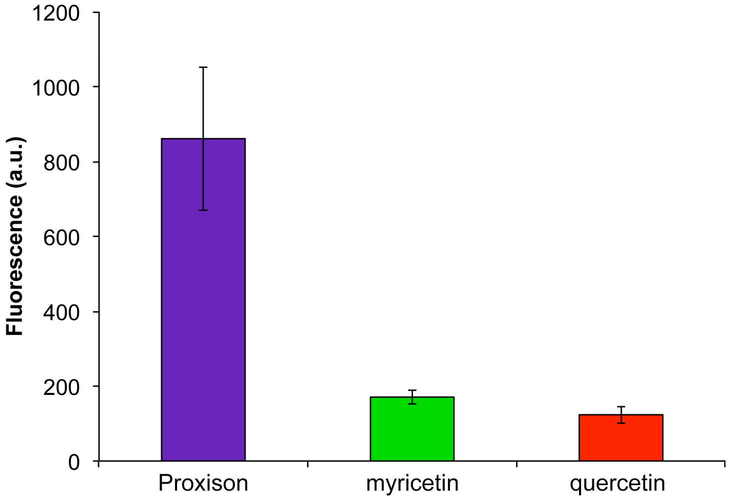

Supplementary Figure 4. Flavonoid antioxidants naturally fluoresce green. The fluorescence of Proxison, myricetin, and quercetin (1 mM) in DMSO was assessed using the FLUOstar microplate reader with excitation 485 nm and emission 520 nm (n=4). Error bars represent the standard error of the mean (SEM).
